# Management of *Stenotrophomonas mediastinitis* after heart transplantation for persistent driveline infection: a case report

**DOI:** 10.1093/jscr/rjaf892

**Published:** 2025-11-14

**Authors:** James Lee West, Charles W Hoopes, Jeremey Walker, Anoma Nellore, Erik J Orozco-Hernandez

**Affiliations:** Division of Cardiothoracic Surgery, Department of Surgery, University of Alabama at Birmingham, 19th Street South, Birmingham, AL 35294, United States; Department of Surgery, Thomas Jefferson University Hospital, 111 South 11th Street, Philadelphia, PA 1907, United States; Division of Cardiothoracic Surgery, Department of Surgery, University of Alabama at Birmingham, 19th Street South, Birmingham, AL 35294, United States; Infectious Diseases, NYU Langone Hospitals, 550 First Avenue, New York, NY 10016, United States; Division of Cardiothoracic Surgery, Department of Surgery, University of Alabama at Birmingham, 19th Street South, Birmingham, AL 35294, United States

**Keywords:** heart transplant, mediastinitis, omentum

## Abstract

We report a 62-year-old male who had a Heartware left ventricular assist device (LVAD) implantation and subsequently developed a persistent driveline infection, culminating in an abscess along the driveline site despite 18 months of antibiotic treatment. His situation was further complicated post-transplant by the onset of mediastinitis, linked to a previously undervalued *Stenotrophomonas maltophilia* infection. This report highlights the potential risks of heart transplantation in patients with unresolved LVAD driveline infections, particularly mediastinitis. The presence of existing driveline infections, combined with the immunosuppressed status of patients, necessitates aggressive surgical measures. Our treatment approach, involving multiple mediastinal washouts, omental flap use, antibiotic bead placement, and an extended post-discharge antibiotic regimen, led to a successful patient outcome. The case emphasizes the importance of thorough antimicrobial coverage for all identified pathogens post-transplantation and advocates for aggressive surgical intervention in managing postoperative complications associated with LVAD driveline infections.

## Introduction

The use of a left ventricular assist device (LVAD) as a bridge to heart transplantation (HTX) is common for the management of end-stage heart failure. However, this therapeutic pathway is not without risks, including the potential for persistent driveline infections. We present a case of a 62-year-old male with ischemic cardiomyopathy who underwent a Heartware (Medtronic, Minneapolis, MN) LVAD implantation while awaiting heart transplantation. The patient developed a chronic driveline infection that persisted despite 18 months of outpatient antibiotic therapy and eventually manifested as an extensive abscess along the driveline site. Our case provides insights into the risks and possible treatment strategies for postoperative complications, specifically mediastinitis, following orthotopic heart transplant in patients with a history of persistent LVAD driveline infections.

## Case report

A 62-year-old male with end-stage heart failure due to ischemic cardiomyopathy underwent Heartware LVAD implantation as a bridge HTX. While waiting for HTX, the patient developed a chronic driveline infection that was originally treated conservatively with outpatient antibiotics. His previous driveline cultures grew multi-drug resistant (extended spectrum beta-lactamase) *Escherichia coli*, *Serratia marcescens*, vancomycin-resistant enterococci, and *Corynebacterium* that were initially managed with daptomycin and fourth generation cephalosporin (cefepime or ceftazidime) therapies. However, after 18 months of antibiotic therapy the patient failed medical treatment and developed a 10 by 2 cm abscess tracking along his driveline site. In the immediate months prior to HTX, he had developed growth of *Stenotrophomonas maltophilia* and this was not covered as it was felt to be more consistent with colonization than a pathogen. Due to his worsening driveline infection, his HTX United Network for Organ Sharing status 1 by exception and 22 months after LVAD implantation the patient underwent HTX. Postoperatively, graft function was satisfactory and immunosuppressive therapy was initiated with methylprednisolone (500 mg) and mycophenolate (1000 mg). Tacrolimus was not given due to the active driveline infection at the time of implantation. The patient’s chest was left open postoperatively also due to the active driveline infection at time of implantation. Negative pressure wound therapy (NPWT) was used. Tacrolimus was started on post-operative day one. On post-operative day two, he returned to the operating room for mediastinal washout and formal chest closure.

**Figure 1:**
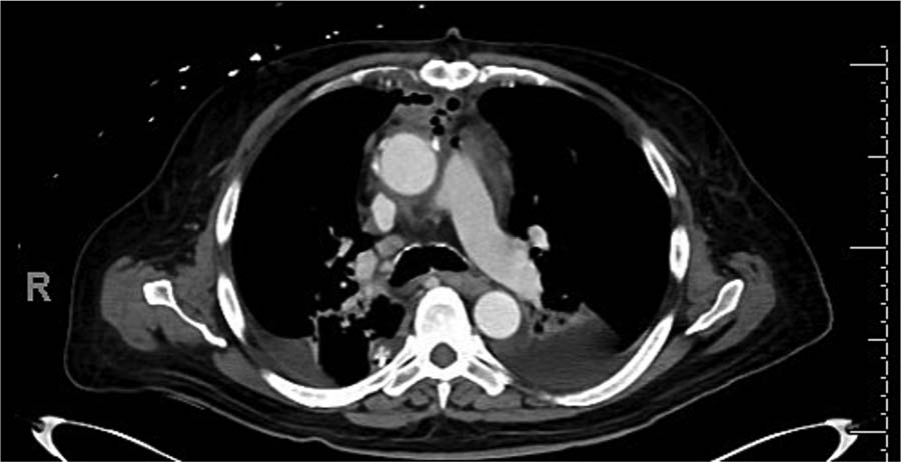
Post operative CT scan concerning for mediastinitis.

**Figure 2:**
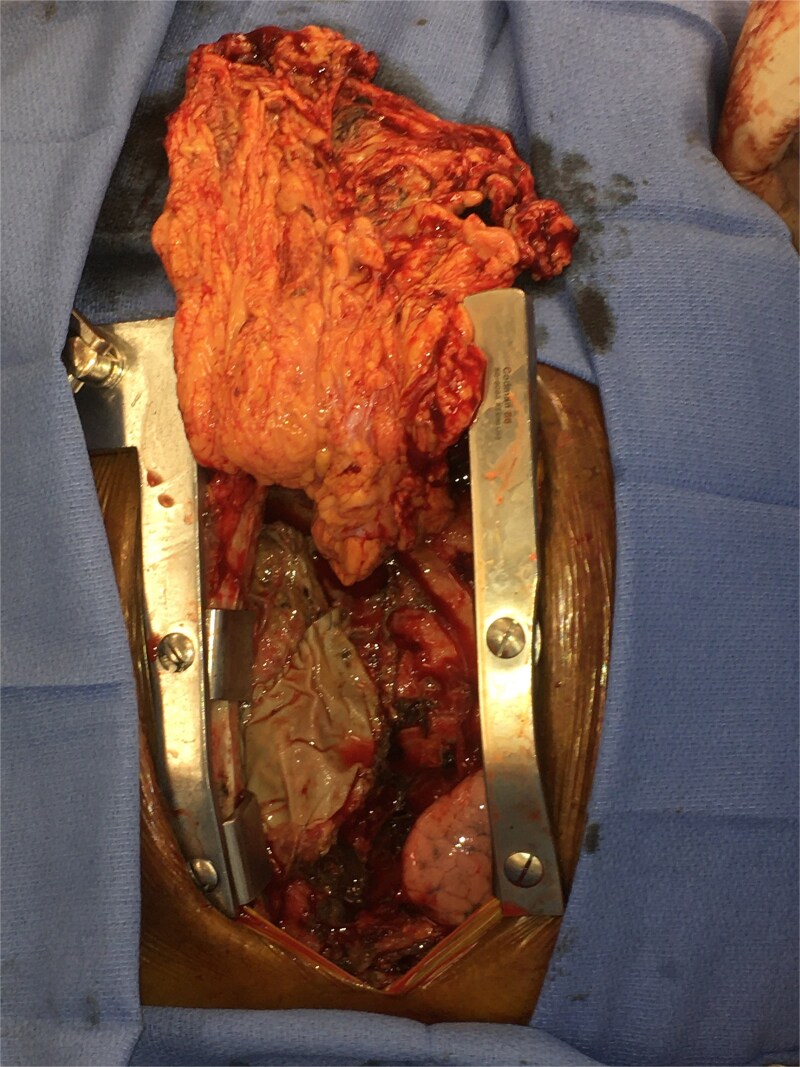
Intra-operative picture of omental flap.

Intravenous (IV) daptomycin and meropenem were continued postoperatively per recommendation of our institution’s infectious disease team and notably this regimen did not cover *S. maltophilia*. On post-operative day 10, the patient had increasing leukocytosis and increased pressor requirements. Computerized tomography (CT) scan was performed that was concerning for mediastinitis (Fig. 1). Blood cultures and pleural fluid cultures from the remaining chest tubes were sent, which eventually grew out *S. maltophilia*. The patient was taken back to the operating room on post-operative day 10. The mediastinum was washed out and the chest was left open again using NPWT. Cultures from the OR also grew out *S. maltophilia*. Two days later (on post-operative day 12), the patient again underwent mediastinal washout and an omental flap (Fig. 2) was mobilized laparoscopically and placed in the mediastinum. The mediastinum was washed out with sodium hypochlorite (0.4%–0.5%) and boric acid (4%) diluted in water, and tobramycin beads were left in the mediastinum. The sternum was closed with wires and the soft tissue was closed with absorbable suture. The skin was closed with staples. The patient was discharged on post-operative day 35 from his transplant with IV daptomycin and IV bactrim for an additional week to finish a 4-week course. He has followed up multiple times in the clinic since his discharge. At present, he is 4 months out from his transplant without any signs of infection and has been off IV antibiotics but remains on Bactrim SS PO daily for prophylaxis, which does provide some coverage of *S. maltophilia*.

## Discussion

Our case report underscores the challenges and potential risks associated with orthotopic heart transplant as a treatment solution for patients presenting with LVAD driveline infections that remain persistent, even following prolonged antibiotic therapy. These conditions likely predispose such patients to postoperative mediastinitis, a risk factor that should be carefully considered during the clinical decision-making process.

The prevalence of deep wound infections such as mediastinitis following HTX is generally reported to fall between 2% and 8% [1–3]. In our particular case, given the presence of an existing driveline infection and the patient’s immunosuppressive state, we elected to take an aggressive surgical approach. This included multiple mediastinal washouts, the placement of antibiotic beads, and the initiation of a long-term post-discharge antibiotic regimen. We also believe the omental flap was crucial to our patient’s success. The omentum, as a robust vascularized tissue, has intrinsic properties that make it capable of reducing dead space, resisting infection, and promoting healing.

Gram negative rod bacterial deep wound infection is uncommon after cardiac surgery. Specifically, mediastinal infection due to *Stenotrophomonas* is even rarer, accounting for only 0.4% of all mediastinal infections in one study [4]. To our knowledge this is the first reported case of successful management of *S. maltophilia* mediastinitis after HTX. It is note-worthy that current treatment recommendations for *S. maltophilia* are evolving and there is emerging literature and new guidance that beta-lactam therapies (i.e. the pre-transplant ceftazidime that this patient received) are unlikely to be effective *in vivo* [5–7].

The current literature lacks comprehensive studies investigating the relationship between relapsed infection and HTX outcomes. Therefore, we believe that a relapsed infection should not be considered a contraindication for HTX. The International Society for Heart and Lung Transplantation consensus statement on LVAD infections recommends that “HTX with associated device removal should be considered in recurrent or relapsed LVAD infection.” [8].

As this case has shown, an active LVAD infection can pose a risk for postoperative mediastinitis after transplantation, particularly in the absence of aggressive antimicrobial coverage of all cultured pathogens. However, an aggressive surgical approach, including the use of an omental flap and long-term effective antibiotic therapy, can help manage this risk effectively.

## References

[1] Abid Q, Nkere UU, Hasan A, et al. Mediastinitis in heart and lung transplantation: 15 years experience. Ann Thorac Surg 2003;75:1565–71. 10.1016/S0003-4975(02)04905-6 12735580

[2] Senechal M, LePrince P, Tezenas du MS, et al. Bacterial mediastinitis after heart transplantation: clinical presentation, risk factors and treatment. J Heart Lung Transplant 2004;23:165–70. 10.1016/S1053-2498(03)00104-9 14761763

[3] Filsoufi F, Rahmanian PB, Castillo JG, et al. Incidence, treatment strategies and outcome of deep sternal wound infection after orthotopic heart transplantation. J Heart Lung Transplant 2007;26:1084–90. 10.1016/j.healun.2007.07.036 18022072

[4] Charbonneau H, Maillet JM, Faron M, et al. Mediastinitis due to gram-negative bacteria is associated with increased mortality. Clin Microbiol Infect 2014;20:O197–202. 10.1111/1469-0691.12369 24520879

[5] Pogue JM, Neelakanta A, Mynatt RP, et al. Carbapenem-resistance in gram-negative bacilli and intravenous minocycline: an antimicrobial stewardship approach at the Detroit Medical Center. Clin Infect Dis 2014;59:S388–93. 10.1093/cid/ciu594 25371515

[6] Khan A, Pettaway C, Dien Bard J, et al. Evaluation of the performance of manual antimicrobial susceptibility testing methods and disk breakpoints for *Stenotrophomonas maltophilia*. Antimicrob Agents Chemother 2023;95:e02631-20. 10.1128/AAC.02631-20 PMC8092892 33558287

[7] Hamdi AM, Fida M, Abu Saleh OM, et al. *Stenotrophomonas* bacteremia antibiotic susceptibility and prognostic determinants: mayo clinic 10-year experience. Open forum Infect Dis 2020;7:ofaa008. 10.1093/ofid/ofaa008 32016126 PMC6988836

[8] Kusne S, Mooney M, Danziger-Isakov L, et al. An ISHLT consensus document for prevention and management strategies for mechanical circulatory support infection. J Heart Lung Transplant 2017;36:1137–53. 28781010 10.1016/j.healun.2017.06.007

